# Leprosy in Brazil in the 21st century: analysis of epidemiological and operational indicators using inflection point regression^⋆^^⋆⋆^

**DOI:** 10.1016/j.abd.2019.09.031

**Published:** 2020-09-18

**Authors:** Carlos Dornels Freire de Souza, João Paulo Silva de Paiva, Thiago Cavalcanti Leal, Gabriel da Silva Urashima

**Affiliations:** Department of Medicine, Universidade Federal de Alagoas, Campus Arapiraca, Arapiraca, AL, Brazil

**Keywords:** Leprosy, Mycobacterium leprae, Time series studies

## Abstract

The objective of this study was to analyze the trend of epidemiological and operational indicators of leprosy in Brazil, from 2001 to 2017. This was a time series study involving nine indicators. The inflection point regression model was used. Decreasing trends were observed for the following: general detection (−4.8%), children under 15 (−3.7%), prevalence (−7.0%), and grade 2/million inhabitants (−3.5%). The proportion of individuals with grade 2 disability showed an upward trend (2.0%) from 2001 as well as contacts examined from 2003 (5.0%). The proportions of cure and of individuals with a degree of disability assessed at the time of the diagnosis and the cure showed a stationary behavior. Although advances are noted, there are still challenges to be overcome.

Leprosy is a neglected tropical disease caused by *Mycobacterium leprae*.^1^ Over the past three decades, the number of cases of the disease has been progressively decreasing. In 2017 alone, 150 countries reported 210,671 new cases of the disease; 80.2% of these were reported by Brazil, India, and Indonesia. That same year, Brazil accounted for 26,875 (92.3%) of the new cases registered in the Americas.^2^

Due to this unfavorable epidemiological scenario, the World Health Organization (WHO) launched the Global Strategy 2016–2020, based on three pillars: a) strengthening government control, coordination, and partnership; b) fighting leprosy and its complications; c) combating discrimination and promoting inclusion.^3^ In this sense, the monitoring of epidemiological indicators is of special relevance for the control of leprosy and the success of the strategies developed.

This study aimed to analyze the temporal evolution of the epidemiological and operational indicators of leprosy in Brazil, from 2001 to 2017.

This was an ecological time series study. Nine epidemiological and operational leprosy indicators in Brazil, obtained from Datasus (http://datasus.saude.gov.br/), were included in the study (Table 1).^4^, ^5^ After collection, the inflection point regression model was used for temporal analysis. The annual percent change (APC) was calculated. The trends were classified as increasing, decreasing, or stationary. A 95% confidence interval and a significance level of 5% were considered. As this study used data in public domain, the need for approval from a research ethics committee was waived.

**Table 1:** Epidemiological and operational indicators selected for the study.

Indicator	Usefulness	Parameters
Detection rate of new leprosy cases in the general population/100,000 inhabitants.	Measure the strength of the endemic morbidity, magnitude, and tendency.	Hyperendemic: ≥40.0/100,000 inhab.
		Very high: 20.00–39.99/100,000 inhab.
		High: 10.00–19.99/100,000 inhab.
		Medium: 2.00–9.99/100,000 inhab.
		Low: <2.00/100,000 inhab.
Detection rate of new leprosy cases in persons under 15 years/100,000 inhabitants.	Measure the strength of the recent transmission of the endemic and its tendency.	Hyperendemic: ≥10.00/100,000 inhab.
		Very high: 5.00–9.99/100,000 inhab.
		High 2.50–4.99/100,000 inhab.
		Medium: 0.50–2.49/100,000 inhab.
		Low: <0.5/100,000 inhab.
Annual leprosy prevalence rate/10,000 inhabitants.	Measure the magnitude of the endemic.	Hyperendemic: ≥20.0/10,000 inhab.
		Very high: 10.0–19.9/10,000 inhab.
		High 5.0–9.9/10,000 inhab.
		Medium: 1.0–4.9/10,000 inhab.
		Low: <1.0/10,000 inhab.
Rate of new leprosy cases with grade 2 physical disability at the time of diagnosis/1 million inhabitants	Assess the deformities caused by leprosy in the general population and compare them with other disabling diseases.	The tendency to reduce the detection rate, accompanied by a reduction in this indicator, characterizes a reduction in the magnitude of the endemic disease.
Proportion of leprosy cases with grade 2 physical disability at the time of diagnosis among the new cases detected and evaluated in the year.	Evaluate the effectiveness of timely and/or early case detection activities.	High: ≥10%.
		Medium: 5%−9.9%.
		Low: <5%.
Proportion of new leprosy cases with any degree of physical disability assessed at diagnosis.	Measure the quality of care in health services.	Good: ≥90%.
		Fair: ≥75%−89.9%.
		Poor: <75%.
Proportion of new leprosy cases with any degree of physical disability assessed at the time of cure.	Measure the quality of care in health services.	Good: ≥90%.
		Fair: ≥75%−89.9%.
		Poor: <75%.
Proportion of examined contacts of new leprosy cases diagnosed in the years of the cohorts.	Measure the services' capacity to carry out surveillance of contacts of new cases of leprosy, increasing the timely detection of new cases.	Good: ≥90.0%.
		Fair: ≥75.0%−89.9%.
		Poor: <75.0%.
Proportion of leprosy cure among new cases diagnosed in the years of the cohorts.	Assess the quality of care and follow-up of newly diagnosed cases until treatment is complete.	Good: ≥90%.
		Fair: ≥75%−89.9%.
		Poor: <75%.

Source: World Health Organization, 2017.^3^

Between 2001 and 2017, 652,764 new cases of leprosy were reported in Brazil. Of these, 41,191 (6.31%) were in individuals under 15 years of age. Since 2013, the detection rates in the general population and in those under 15 years showed a downward trend (APC = −5.9% and −5.0%; respectively). When the complete period was considered, the percentages of reduction were smaller (−4.8% and −3.7%, respectively). Despite the advances, in 2017 the endemic was classified as high in the general population (12.94/100,000) and in children under 15 years old (3.72/100,000). The prevalence rate, in turn, has shown a linear trend of reduction since 2001 (APC = −7.0%), decreasing from 3.99 to 1.35/10,000 inhabitants (Fig. 1 and Table 2).

**Figure 1:**
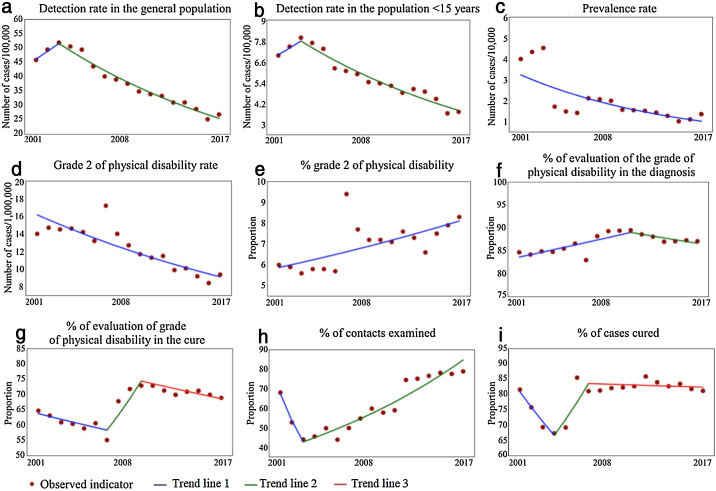
Epidemiological and operational indicators of leprosy in Brazil, 2001−2017.

**Table 2:** Trend of leprosy epidemiological and operational indicators in Brazil, 2001–2017.

Indicator	Period	APC (95% CI)	*p*-value	Trend
Detection rate of new leprosy cases in the general population/100,000 inhabitants	2001−2003	3.8 (−8.8 to 18.1)	0.5	Stationary
	2003−2017	−5.9 (−6.5 to −5.4)	<0.001	Descending
	2001−2017	−4.8 (−6.2 to −3.3)	<0.001	Descending
Detection rate of new leprosy cases in persons under 15 years/100,000 inhabitants	2001−2003	5.5 (−12.1 to 26.7)	0.5	Stationary
	2003−2017	−5.0 (−5.8 to −4.2)	<0.001	Descending
	2001−2017	−3.7 (−5.8 to −1.6)	<0.001	Descending
Annual leprosy prevalence rate/10,000 inhabitants	2001−2017	−7.0% (−9.7 to −4.2)	<0.001	Descending
Rate of new leprosy cases with grade 2 physical disability at the time of diagnosis/1 million inhabitants	2001−2017	−3.5 (−4.5 to −2.5)	<0.001	Descending
Proportion of leprosy cases with grade 2 physical disability at the time of diagnosis among the new cases detected and evaluated in the year	2001−2017	2.0 (0.8 to 3.3)	<0.001	Ascending
Proportion of new leprosy cases with any degree of physical disability assessed at diagnosis	2001−2011	0.6 (0.3 to 1.0)	<0.001	Ascending
	2011−2017	−0.4 (−1.2 to 0.3)	0.2	Stationary
	2001−2017	0.2 (−0.1 to 0.6)	0.2	Stationary
Proportion of new leprosy cases with any degree of physical disability assessed at time of cure	2001−2007	−1.5 (−2.3 to −0.6)	<0.001	Descending
	2007−2010	8.4 (3.1 to 14.0)	<0.001	Ascending
	2010−2017	−1.1 (−1.8 to −0.5)	<0.001	Descending
	2001−2017	0.5 (−0.4 to 1.4)	0.3	Stationary
Proportion of examined contacts of new leprosy cases diagnosed in the years of the cohorts	2001−2003	−20.4 (−35.5 to −1.7)	<0.001	Descending
	2003−2017	5.0 (4.0 to 6.1)	<0.001	Ascending
	2001−2017	1.4 (−1.1 to 4.0)	0.3	Stationary
Proportion of leprosy cure among new cases diagnosed in the years of the cohorts	2001−2004	−6.1 (−8.8 to −3.6)	<0.001	Descending
	2004−2007	7.8 (2.1 to 13.9)	3.1	Descending
	2007−2017	−0.1 (−0.6 to 0.3)	0.5	Stationary
	2001−2017	0.1 (−0.9 to 1.1)	0.8	Stationary

95% CI, 95% confidence interval; APC, annual percent change.

Among the indicators related to the presence of physical disability, the rate of new leprosy cases with grade 2 physical disability at diagnosis has shown a downward trend since 2001 (APC = −3.5%), decreasing from 14.00 to 9.39/1 million inhabitants. In turn, the proportion of individuals diagnosed with grade 2 showed a significant growth trend (APC = 2.0%), going from 6.0% in 2001 to 8.3% in 2017 (Fig. 1 and Table 2).

The proportions of individuals assessed at the time of the diagnosis and the cure presented a stationary temporal pattern in the studied period. In all years of the time series, the proportion of subjects evaluated was considered regular (75%–89.9%) at the time of diagnosis and precarious (<75%) at the time of discharge due to cure (Fig. 1 and Table 2).

The proportion of contacts examined showed a growth trend since 2003 (APC = 5.0%), going from 43.9% in 2003 to 78.9% in 2017, thus being considered regular. In turn, the proportion of individuals cured showed a steady trend in the period, with 81.6% in 2001 and 81.2% in 2017, thus being considered regular (Fig. 1 and Table 2).

Even considering the advances in the fight against leprosy, the findings show important challenges to be overcome by Brazil. The first one is the high annual percentages of reduction in general detection (−4.8%), detection in children under 15 years old (−3.7%), and detection of grade 2 disability (−7.0%). As this is a chronic disease with slow evolution and difficult control, such sharp annual reductions should be viewed with concern. For example, in 2005, over 49,000 cases of the disease were registered in the general population; the following year, slightly over 43,000. Brazilian studies point to the high hidden prevalence of the disease in Brazil, which has prevented the identification of the real number of patients in the country.^5^ In the present study, the increase in the proportion of grade 2 cases at diagnosis reinforces this underdiagnosis hypothesis.^6^, ^7^

The second Brazilian challenge is the need to qualify the surveillance system. There is a lack of qualified outpatient clinics and personnel for the proper monitoring of cases, which compromises the assessment of the degree of disability, the monitoring of neural functions, the management of reactions, and the proper examination of contacts, fundamental tools for interrupting the transmission chain of this disease.^8^, ^9^, ^10^

Based on the present findings, the authors advocate the need for the development of plans and strategies that would allow early diagnosis of the disease and favor the systematic monitoring of patients, with emphasis on early diagnosis and prevention of physical disabilities.

## Financial support

None declared.

## Authors’ contributions

Carlos Dornels Freire de Souza: Statistical analysis; approval of the final version of the manuscript; elaboration and writing of the manuscript; obtaining, analyzing, and interpreting the data; effective participation in research orientation; critical review of the literature; critical review of the manuscript.

João Paulo Silva de Paiva: Statistical analysis; approval of the final version of the manuscript; elaboration and writing of the manuscript; obtaining, analyzing, and interpreting the data; critical review of the literature; critical review of the manuscript.

Thiago Cavalcanti Leal: Statistical analysis; approval of the final version of the manuscript; elaboration and writing of the manuscript; obtaining, analyzing, and interpreting the data; critical review of the literature; critical review of the manuscript.

Gabriel da Silva Urashima: Statistical analysis; approval of the final version of the manuscript; elaboration and writing of the manuscript; obtaining, analyzing, and interpreting the data; critical review of the literature; critical review of the manuscript.

## Conflicts of interest

None declared.

## References

[1] Cruz R.C.S., Buhrer-Sékula, Penna M.L.F., Penna G.O., Talhari S. (2017). Leprosy: current situation, clinical and laboratory aspects, treatment history and perspective of the uniform multidrug therapy for all patients. An Bras Dermatol..

[2] World Health Organization (2017). Global leprosy update, 2017: reducing the disease burden due to leprosy. Wkly Epidemiol Rec..

[3] World Health Organization (2017). Global leprosy update, 2016: accelerating reduction of disease burden. Wkly Epidemiol Rec..

[4] Ministério da Saúde. Secretaria de Vigilância em Saúde. Departamento de Vigilância das Doenças Transmissíveis. Diretrizes para vigilância, atenção e eliminação da Hanseníase como problema de saúde pública: manual técnico-operacional [Internet]. Brasília: Ministério da Saúde; 2016 [cited 2018 Oct 19]. Available from: http://www.saude.pr.gov.br/arquivos/File/Manual_de_Diretrizes_Eliminacao_Hanseniase.pdf.

[5] Departamento de Informática do Sistema Único de Saúde. Casos de Hanseníase (SINAN). [cited 2019 Mar 28]. Available from: http://www2.datasus.gov.br/DATASUS/index.php?area=0203&id=31032752.

[6] Salgado C.G., Barreto J.G., Silva M.B., Goulart I.M.B., Barreto J.A., Nery J.A. (2018). Are leprosy case numbers reliable?. Lancet Infect Dis..

[7] Bernardes-Filho F., Paula N.A., Leite M.N., Abi-Rached T.L.C., Vernal S., Silva M.B.D. (2017). Evidence of hidden leprosy in a supposedly low endemic area of Brazil. Mem Inst Oswaldo Cruz..

[8] Souza C.D.F., Santos F.G.B., Marques C.S., Leal T.C., Paiva J.P.S., EMCF Araújo (2018). Spatial study of leprosy in Bahia, Brazil, 2001-2012: an approach based on the local empirical Bayesian model. Epidemiol Serv Saúde..

[9] Barbieri R.R., Sales A.N., Hacker M.A., Nery J.A.C., Dupre N.D., Machado A.M. (2016). Impact of a reference center on leprosy control under a decentralized Evidence of hidden leprosy in a supposedly low endemic area of Brazil public health care policy in Brazil. PLoS Negl Trop Dis..

[10] Souza C.D.F., Luna C.F., Magalhães M.A.F.M. (2019). Spatial modeling of leprosy in the State of Bahia and its social determinants: a study of health inequities. Ann Bras Dermal..

